# An Experience With an Exoscope System (ORBEYE) for Surgery for Tarsal Tunnel Syndrome: A Case Report

**DOI:** 10.7759/cureus.28045

**Published:** 2022-08-15

**Authors:** Isamu Miura, Kotaro Kohara, Takakazu Kawamata

**Affiliations:** 1 Department of Neurosurgery, Tokyo Women’s Medical University, Tokyo, JPN; 2 Department of Neurosurgery, Tokyo Women's Medical University, Tokyo, JPN

**Keywords:** posterior tibial nerve, posterior tibial artery, peripheral entrapment neuropathy, tarsal tunnel syndrome, exoscope

## Abstract

Surgery for peripheral entrapment neuropathy aims to decompress the affected nerve and optimize the visualization of anatomical details during surgery. This paper describes our experience using the ORBEYE exoscope (Olympus) during surgery for tarsal tunnel syndrome (TTS).

The patient was a 70-year-old male with complaints of bilateral pain and numbness on the plantar surface of the bilateral soles and medial halves of both lower limbs. He was diagnosed with idiopathic TTS with the American Orthopedic Foot and Ankle Score (AOFAS) of 20/100. Surgery for the right foot was performed under local anesthesia with the patient’s body in the lateral position. All procedures were performed using the ORBEYE exoscope view. The posterior tibial artery (PTA) was transposed, and the flexor retinaculum was reconstructed between the PTA and posterior tibial nerve. Indocyanine green (ICG) video angiography confirmed the absence of PTA flow disturbance. One month after the first operation, left foot surgery was performed. Three months later, the AOFAS had improved from 20/100 to 50/100.

The ORBEYE exoscope is useful in TTS treatment and represents a feasible and comfortable technique for entrapment neuropathy surgery. In addition, ICG capability is an effective tool for confirming blood flow in PTA after transposition.

## Introduction

The performance of surgical interventions in a small anatomical structure, such as the peripheral nerves, requires a magnified view of anatomical details. The use of a conventional surgical microscope during surgery for entrapment neuropathies such as carpal tunnel syndrome has been reported [1]. Recently, exoscopes, including ORBEYE (OLYMPUS, Tokyo, Japan), have been introduced to the field of neurosurgery. The ORBEYE exoscope has several relevant differences from a traditional microscope. During surgery, the surgeon operates while viewing surgical field images on a monitor. The standalone optical system uses cameras with very high resolutions (3,840 × 2,160 pixels), and the position, zoom, and focus can be directly controlled by hand or by a foot switch system [2,3]. Images are presented in 3D on a monitor with specialized light eyeglasses. Exoscopic surgery provides high-quality images with sufficient magnification, zoom, and luminance [2]. In addition, infrared sensors used for indocyanine green (ICG) video angiography and blue light for 5-aminolevulinic acid fluorescence are available. These advantages have been reported for cranial, spinal, and otologic surgery [3-5]. However, most reports of exoscopic procedures for peripheral nerves have focused on interventions for peripheral tumors [2,6], and evidence for the use of a 4K-3D exoscopic system in surgery for entrapment neuropathy has been limited [3]. Herein, we report a successful surgical case employing the ORBEYE in a patient with tarsal tunnel syndrome (TTS). We have added a review of the literature on ORBEYE exoscope and surgery for peripheral entrapment neuropathy.

## Case presentation

**Figure 1:**
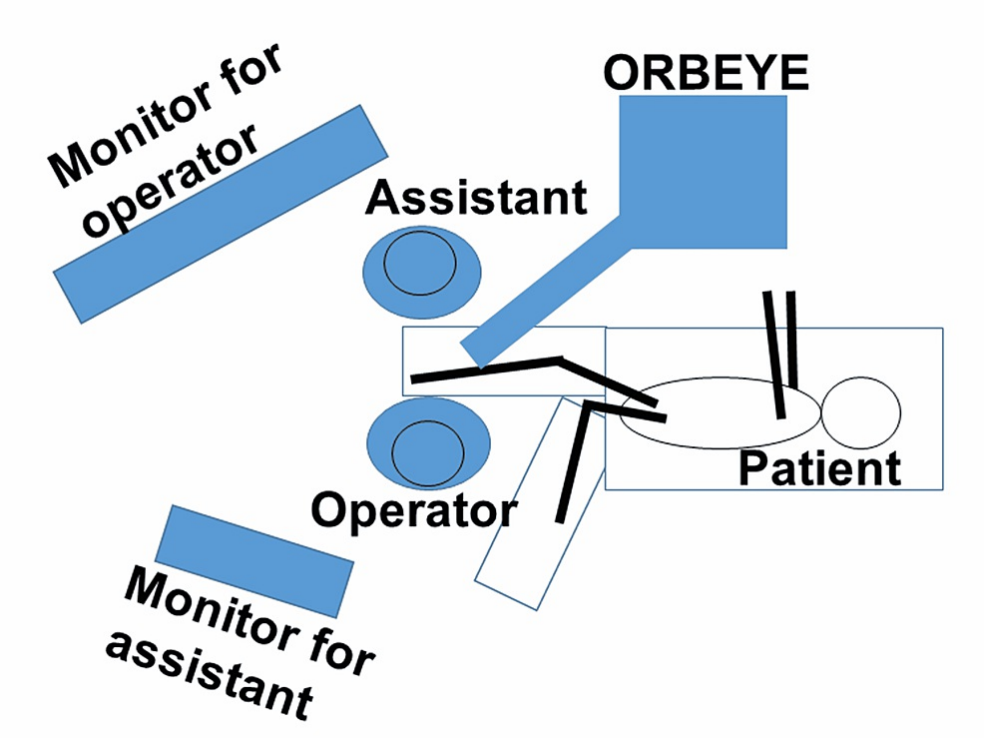
Schematic drawing of the surgical setting in the operating room showing the patient is placed in the right lateral position for surgery on the right foot.

**Figure 2:**
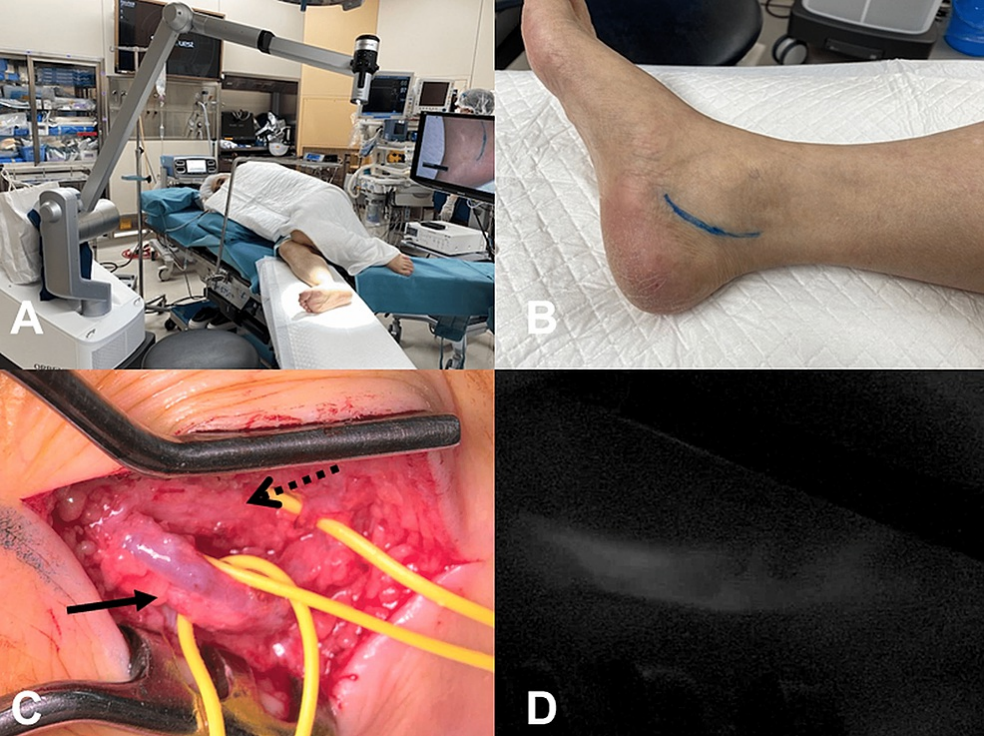
Preoperative operating room setting and intraoperative view (A) Preoperative operating room setting with the patient placed in the right lateral position and the exoscope prepared. (B) Skin incision along the posterior tibial artery. (C) Intraoperative view. The black arrow shows the posterior tibial artery, and the dotted arrow shows the posterior tibial nerve. (D) Indocyanine green video angiography showing the flow of the posterior tibial artery.

The patient was a 70-year-old male with a history of cardiac angina, hypertension, and diabetes mellitus. He presented to Tokyo Women’s Medical University Hospital with complaints of pain and numbness bilaterally on the plantar surface of the bilateral soles and medial halves of both lower limbs, which had appeared five years before his visit. The Tinel-like sign of the bilateral ankles was positive. Lidocaine injection into the right tarsal tunnel diminished paresthesia and pain. Therefore, the patient was diagnosed with idiopathic TTS. The American Orthopedic Foot and Ankle Score (AOFAS) was 20/100. Surgery for the right foot was performed under local anesthesia with the patient in the lateral position (Figures 1, 2A). The operator viewed the images on the 4K monitor and the assistant viewed the images on the sub-monitor. All procedures performed by the operator were conducted using the ORBEYE view. After a 5-cm skin incision was made on the flexor retinaculum (Figure 2B), the flexor retinaculum was resected, and the posterior tibial artery (PTA) was decompressed from the posterior tibial nerve (PTN) (Figure 2C). The flexor retinaculum was sutured between the PTA and the PTN. ICG was used to confirm the absence of PTA flow disturbance (Figure 2D). We encountered no intraoperative or postoperative complications and the symptoms improved postoperatively. One month after the first surgery, surgery for the left foot was performed with all procedures identical to those performed during the first surgery. There were no complications such as wound infection or neurological deterioration. The surgical durations for the right and left feet were 51 and 52 min, respectively. At the three-month follow-up, the AOFAS had improved from 20/100 to 50/100, and the patient reported being able to maintain a normal physical routine.

## Discussion

Surgical procedures for peripheral entrapment neuropathy under a surgical microscope and endoscope have been reported; a surgical microscope is used to improve the optical magnification and illumination of the surgical field [7-9]. The ORBEYE exoscope is a recently introduced intraoperative optical system that employs a small orientable camera equipped with an arm. The image quality is notably higher than that of conventional microscopes [2,3,10]. By offering 4K-3D images with extremely high definition, zoom, and wider focal distance, the ORBEYE exoscope enables detailed anatomical visualization, which is beneficial for procedures requiring high magnification, including minimally invasive interventions for small compartments required to treat TTS [2,3,10]. For TTS, the PTA and PTN are tightly combined in the tarsal tunnel; therefore, the treatment of idiopathic TTS involves dissection of the nerve and the artery or venous complex [9]. Dissection of the PTA or vascular complex from PTN should be performed carefully because of the risk of nerve or vascular associated with adhesion between the PTA and PTN, and PTA and PTN sometimes have thin branches [11]. Greater magnification of the ORBEYE exoscope may help to prevent these injuries.

Another merit of the ORBEYE exoscope is that it is superior to microscopes in terms of ergonomic features [2,12]. Improved surgeon ergonomics during neurosurgical procedures have been reported by several authors [2,3]. The camera angle in the ORBEYE exoscope can be changed widely. The exoscope also allows a neutral cervical posture by placing the monitor at eye level and in front of the surgeon. An upright head position was maintained during the operation. This is aimed at relieving the surgeon of musculoskeletal pain [2]. The superiority of the ergonomics of the exoscope over that of conventional microscopy should be further evaluated.

The ORBEYE exoscope has an infrared sensor that can be used with ICG. In TTS surgery, transposition of the PTA is useful; however, it may cause poor blood flow in the PTA. We can confirm the blood flow of the transposed PTA using ICG with the ORBEYE exoscope, as the efficacy of ICG in confirming the presence of blood flow in the transposed PTA has been reported by Fujihara et al. [11].

The use of the ORBEYE has achieved a superior educational value [3,13]. The exoscope provides surgeons, assistants, and everyone who watches the 55-inch or 31-inch external monitor using polarized glasses with the high-resolution 4K-3D images of the procedure, starting with skin incision. Residents and students attending surgery may enjoy the surgical field in detail and surgical techniques. The use of the exoscope was considered helpful in training surgeons to perform surgical procedures [13].

The ORBEYE exoscope may not be suitable for all surgeries. Surgeons tend to switch from the exoscope to the conventional microscope when approaching deep or vulnerable structures, because of stereopsis although stereopsis is reported to be at least equivalent [2,3,14]. TTS surgery requires a relatively shallow operative field, and stereopsis does not matter.

## Conclusions

Compared with a traditional microscope, the ORBEYE exoscope may lead to improved visualization of the surgical field and increased comfort and safety in neurosurgery. It can provide high-quality images with high magnification and luminance for surgery for entrapment neuropathy. In addition, the ORBEYE exoscope has an infrared sensor for ICG, which is effective in monitoring real-time blood flow and confirming blood flow in the PTA after transposition in TSS operation.

## References

[1] Shapiro S (1995). Microsurgical carpal tunnel release. Neurosurgery.

[2] Rösler J, Georgiev S, Roethe AL (2022). Clinical implementation of a 3D4K-exoscope (Orbeye) in microneurosurgery. Neurosurg Rev.

[3] Murai Y, Sato S, Yui K (2019). Preliminary clinical microneurosurgical experience with the 4K3- Dimensional Microvideoscope (ORBEYE) system for microneurological surgery: observation study. Oper Neurosurg (Hagerstown).

[4] Kanzaki S, Takahashi S, Toda M, Yoshida K, Ogawa K (2021). Pros and cons of the exoscope for otologic surgery. Surg Innov.

[5] Kwan K, Schneider JR, Du V (2019). Lessons learned using a high-definition 3-dimensional exoscope for spinal surgery. Oper Neurosurg (Hagerstown).

[6] Vetrano IG, Acerbi F, Falco J, D'Ammando A, Devigili G, Nazzi V (2020). High-definition 4K 3D exoscope (Orbeyetm) in peripheral nerve sheath tumor surgery: a preliminary, explorative, pilot study. Oper Neurosurg (Hagerstown).

[7] Rose EH, Norris MS, Kowalski TA, Lucas A, Flegler EJ (1991). Palmaris brevis turnover flap as an adjunct to internal neurolysis of the chronically scarred median nerve in recurrent carpal tunnel syndrome. J Hand Surg Am.

[8] Orhurhu V, Orman S, Peck J (2020). Carpal tunnel release surgery- a systematic review of open and endoscopic approaches. Anesth Pain Med.

[9] Kohno M, Takahashi H, Segawa H, Sano K (2000). Neurovascular decompression for idiopathic tarsal tunnel syndrome: technical note. J Neurol Neurosurg Psychiatry.

[10] Takahashi S, Toda M, Nishimoto M (2018). Pros and cons of using ORBEYE™ for microneurosurgery. Clin Neurol Neurosurg.

[11] Fujihara F, Isu T, Kim K (2020). Artery transposition using indocyanine green for tarsal tunnel decompression. World Neurosurg.

[12] Shimizu T, Toyota S, Nakagawa K, Murakami T, Mori K, Kishima H, Taki T (2021). Retrosigmoid approach in the supine position using Orbeye: a consecutive series of 14 cases. Neurol Med Chir (Tokyo).

[13] Ricciardi L, Chaichana KL, Cardia A, Stifano V, Rossini Z, Olivi A, Sturiale CL (2019). The exoscope in neurosurgery: an innovative "point of view". A systematic review of the technical, surgical and educational aspects [PREPRINT]. World Neurosurg.

[14] Sack J, Steinberg JA, Rennert RC, Hatefi D, Pannell JS, Levy M, Khalessi AA (2018). Initial experience using a high-definition 3-dimensional exoscope system for microneurosurgery. Oper Neurosurg (Hagerstown).

